# Implementation of a quantum cascade laser-based gas sensor prototype for sub-ppmv H_2_S measurements in a petrochemical process gas stream

**DOI:** 10.1007/s00216-016-9923-z

**Published:** 2016-09-17

**Authors:** Harald Moser, Walter Pölz, Johannes Paul Waclawek, Johannes Ofner, Bernhard Lendl

**Affiliations:** 1 Institute of Chemical Technologies and Analytics, Vienna University of Technology, 1060 Vienna, Austria; 2 OMV R&M GmbH, 2320 Schwechat, Austria

**Keywords:** Infrared laser spectroscopy, Quantum cascade lasers, Laser sensor, Hydrogen sulfide, Methane, HDS monitoring

## Abstract

The implementation of a sensitive and selective as well as industrial fit gas sensor prototype based on wavelength modulation spectroscopy with second harmonic detection (2*f*-WMS) employing an 8-μm continuous-wave distributed feedback quantum cascade laser (CW-DFB-QCL) for monitoring hydrogen sulfide (H_2_S) at sub-ppm levels is reported. Regarding the applicability for analytical and industrial process purposes aimed at petrochemical environments, a synthetic methane (CH_4_) matrix of up to 1000 ppmv together with a varying H_2_S content was chosen as the model environment for the laboratory-based performance evaluation performed at TU Wien. A noise-equivalent absorption sensitivity (NEAS) for H_2_S targeting the absorption line at 1247.2 cm^−1^ was found to be 8.419 × 10^−10^ cm^−1^ Hz^−1/2^, and a limit of detection (LOD) of 150 ppbv H_2_S could be achieved. The sensor prototype was then deployed for on-site measurements at the petrochemical research hydrogenation platform of the industrial partner OMV AG. In order to meet the company’s on-site safety regulations, the H_2_S sensor platform was installed in an industry rack and equipped with the required safety infrastructure for protected operation in hazardous and explosive environments. The work reports the suitability of the sensor prototype for simultaneous monitoring of H_2_S and CH_4_ content in the process streams of a research hydrodesulfurization (HDS) unit. Concentration readings were obtained every 15 s and revealed process dynamics not observed previously.

## Introduction

Sensitive and selective detection of hydrogen sulfide (H_2_S) is essential for production control and environmental monitoring purposes in the field of petrochemical, paper and pulp, or biotechnological processes. Despite a variety of online monitoring options for gaseous hydrogen sulfide, its reliable quantitative and selective determination still remains challenging in the field of chemical sensors [1–3].

Hydrodesulfurization (HDS) is one of the most important process operations in the modern petroleum refining industry and is receiving increased attention due to the stringent environmental regulations on the sulfur content in transport fuels (gasoline, diesel, and jet fuel). HDS is a catalytic process by which sulfur-containing impurities are removed from crude petroleum feedstocks and fuels during hydrogen exposition in the presence of cobalt-promoted molybdenum (CoMo) or nickel-promoted molybdenum (NiMo) catalysts and formation of H_2_S [4]. The HDS process is of both industrial and environmental importance: Firstly, the sulfur-containing impurities in hydrocarbon fuels are effective catalyst poisons, preventing untreated crude feedstocks from being used for subsequent chemical transformations, and secondly, the severe environmental impact is stemming from the emitted sulfur oxides during combustion and their contribution to acid rain [5].

Since its first operational demonstration in 1994 [6], the quantum cascade laser (QCL) advanced to a powerful and reliable spectroscopic source of coherent light covering the mid-infrared (MIR) and terahertz spectral region for sensitive detection of molecular species on their fundamental vibrational bands and rendered laser-based absorption spectroscopy a powerful tool for industrial gas sensing [6–9]. The high-quality implications encompassing stringent single-mode emission and superior wavelength stability as required for industrial trace gas sensing are met by distributed feedback (DFB)-type QCLs [10]. Commercial DFB-type QCLs are configured as edge-emitting ridge lasers and conventionally housed in standardized, thermo-electrically (TE) stabilized semiconductor packages. These lasers can be tuned by either modulation of the injection current and/or changing the temperature of the gain medium. The resulting tuning range is limited to a few wavenumbers only, and thus, typically one or two analytes can be spectroscopically targeted by a given DFB-type QCL.

Different approaches for QCL-based quantitative gas phase spectroscopy have been demonstrated, and the technical details were recently reviewed [11]. The included cavity-enhanced absorption spectroscopy [12], quartz-enhanced photoacoustic spectroscopy [13, 14], and open-path setups [15–17] were all successfully applied to industrial and environmental monitoring. The “golden standard” for QCL-based trace gas measurements in the MIR spectral region is established on absorbance measurements in multipass-reflection cells [18]. While special cell types were successfully tested [19], the basic cell type for laser spectroscopy is the so-called Herriott cell [20], as the effective optical interaction pathlength can easily reach up to several tens of meters. Together with phase-sensitive detection techniques, such as wavelength modulation spectroscopy (WMS) [21, 22], the generally dominating *1*/*f* electronic noise can be drastically minimized and generally high detection sensitivities can be achieved [23].

Recently, an FTIR-based sensing approach of H_2_S, by using a UV-assisted conversion of H_2_S into the much more pronounced responding SO_2_, is presented in [2]. QCL-based H_2_S monitoring in N_2_ with a pulsed source of 1253.5 cm^−1^ is demonstrated in [24].

In the present work, a sensitive, selective, and industrial fit gas sensor setup based on second harmonic wavelength modulation spectroscopy (2*f*-WMS) employing an 8-μm continuous-wave distributed feedback quantum cascade laser (CW-DFB-QCL) was developed and implemented for detecting H_2_S at sub-ppm levels in petrochemical process gas streams. Due to the possibility of molecular discrimination between H_2_S and methane (CH_4_) ro-vibrational transitions, a simultaneous detection of these two analytes could be implemented. On-site tests of the sensor were performed at the research hydrogenation platform of OMV AG.

## Materials and methods

### Process analytical importance of H_2_S in the HDS process

In an industrial petrochemical unit, the HDS reaction takes place in a fixed-bed reactor at temperatures ranging from 300 to 450 °C and pressures ranging from 30 to 130 bar in the presence of a catalyst typically consisting of an alumina base impregnated with cobalt or nickel and molybdenum (CoMo, NiMo).

1 $$ ``\left[R-S\right]"+\frac{3}{2}{\mathrm{H}}_2\underset{300-450\kern0.5em {}^{\circ}\mathrm{C},\ 30-130\kern0.5em \mathrm{bar}}{\overset{\mathrm{CoMo},\ \mathrm{NiMo}}{\to }}``\left[R-H\right]"+{\mathrm{H}}_2\mathrm{S} $$

For the production of ultra-low sulfur fuel feedstocks (<10 ppmw S), more than 99 % of the sulfur compounds present in the feedstock ought to be removed during this catalytic hydrotreating process. The shift from normal to ultra-deep desulfurization is a very challenging technical problem, as many factors such as the catalysts, process parameters, feedstock source and quality, reactivities of the present sulfur compounds, and inhibition effects of nitrogen compounds, aromatics, and predominantly H_2_S can have significant influences on the degree of desulfurization of the feedstocks [4, 5, 25–28].

As the HDS reaction pathway proceeds mainly via the two parallel routes—direct desulfurization (DDS) and hydrogenation (HYD)—the poisoning effects of the main inhibitor H_2_S have been found to be different for the two routes. In particular, H_2_S is a strong inhibitor for sulfur removal via the DDS route, but it only has a minor effect on HYD route. Since H_2_S is the by-product of HDS reaction, its presence in a hydrotreating reactor is unavoidable. Susceptibilities to H_2_S poisoning are different for the various types of catalysts. Moreover, at low partial pressures, H_2_S also plays a beneficial role in maintaining the sulfide state of the CoMo and NiMo catalysts and may enhance hydrogenation. H_2_S partial pressure also has a strong influence on the susceptibility of the employed NiMo and CoMo catalysts to H_2_S poisoning and thus has a promotional effect on the overall HDS reaction.

Significant improvements in HDS catalysts and reactor design have been made, and optimum operating strategies have been developed to minimize the inhibition effects of H_2_S and other inhibitory compounds and to enhance the removal of the last traces of refractory sulfur compounds [5, 25–28].

In this context, a selective and sensitive H_2_S sensor with a fast response time is of utmost analytical process value in order to maintain the HDS unit under optimum operational parameters. Gas chromatography (GC) is considered the standard petrochemical analytical technique as it is often used for measuring the entire composition of a sample down to ppmv concentrations while also measuring the majority component up to 100 %v. However, to reach sub-ppmv and ppbv measurements along with high-percent level measurements, a GC usually requires separate injection and column switching techniques, turning it into a complex and expensive analyzer. In addition, GC cycle times are usually in the range of 5–15 min, depending on the application, and thus, the concentration data is rather periodic than continuous. A laser-based system for direct and selective measurement as possible when using QCLs can therefore be the preferred technique for applications where continuous measurements with a fast sensor response are required. In terms of sensitivity and dynamic range, QCL-based sensors can offer better performance than GC while response times are typically <30 s [29–31].

### The H_2_S and CH_4_ spectral line system in the 8-μm region

In order to assess the applicability for selective and sensitive H_2_S measurements for analytical and industrial process purposes, a synthetic CH_4_ matrix as a very strong absorbent gas species was chosen as the model environment. Based on CH_4_ interference-free H_2_S lines and commercially available CW-DFB-QCLs, the 8-μm MIR spectral region between 1250 and 1245 cm^−1^ was chosen for the measurements of H_2_S and CH_4_. This region is characterized by the overlapping of the *ν*_2_(*A*_1_) bending mode transition of H_2_S (*C*_2v_ symmetry) and the *ν*_4_(*F*_2_) bending mode of CH_4_ (*T*_d_ symmetry) [32]. Further, a Coriolis coupling of the *ν*_4_(*F*_2_) and the *ν*_2_(*E*) is present, causing the presence of *ν*_4_-*ν*_2_-coupled ro-vibrational transitions [32, 33]. For the selection of the most suitable range for the spectral detection of H_2_S, reference spectra were calculated [34] based on the HITRAN [35] database.

In this work, the spectral region (1250–1245 cm^−1^) corresponding to the ro-vibrational transitions of the *ν*_2_ bending mode of H_2_S listed in Table 1 was considered for the laser tuning and characterization measurements. The rotational levels of H_2_S as a three-dimensional asymmetric top rotator with three different reciprocal moments of inertia are labeled by the three standard quantum numbers: *J*, *K*_a_, and *K*_c_ [36].

**Table 1:** Main ro-vibrational transitions of the *ν*_2_ bending mode of H_2_S in the spectral region (1250–1245 cm^**−**1^)

Wavenumber (cm^−1^)	Linestrength (cm mol^−1^)	LRVS_upper_^a^[*J*′ *K*_a_′ *K*_c_′]	LRVS_lower_^a^[*J*″ *K*_a_″ *K*_c_″]	Remarks
1245.84424	9.57*E*−23	[8 3 5]	[8 2 6]	Low linestrength
1246.07617	2.88*E*−22	[8 4 5]	[8 3 6]	CH_4_ interference
1247.19629	6.75*E*−22	[5 1 4]	[4 2 3]	Accessible
1247.53418	2.29*E*−22	[5 2 4]	[4 1 3]	Accessible
1248.37695	9.68*E*−22	[3 3 0]	[2 2 1]	CH_4_ interference
1249.15698	1.58*E*−22	[9 4 5]	[9 3 6]	Low linestrength
1249.21899	3.79*E*−22	[7 1 6]	[7 0 7]	Medium linestrength
1249.22071	1.26*E*−22	[7 2 6]	[7 1 7]	Low linestrength

^a^Quantum numbers of upper and lower local ro-vibrational state (LRVS)

### Laser characterization and absorption line selection

For spectral H_2_S assessment, a collimated CW-DFB-QCL in a high-heat load (HHL) package (sbcw 5704, Alpes Lasers) emitting at ∼8.0 μm was employed, generating up to 35 mW of coherent optical radiation. In order to perform selective and sensitive H_2_S 2*f*-WMS measurements, the strongest absorption lines in the 1250–1245 cm^−1^ region were targeted. The single-mode operation and the time-resolved spectral behavior of the DFB-QCL in the 1250–1245 cm^−1^ spectral range were investigated using a step-scan-enabled Fourier transform IR spectrometer (Bruker Vertex 80v, Bruker Optics, Germany) with a spectral resolution of 0.075 cm^−1^ and temporal resolution of 2 ns [37, 38]. A liquid nitrogen (LN_2_)-cooled mercury-cadmium-telluride (MCT) detector with a response time <2 ns (Kolmar Technologies, USA) served as the infrared detector.

**Fig. 1:**
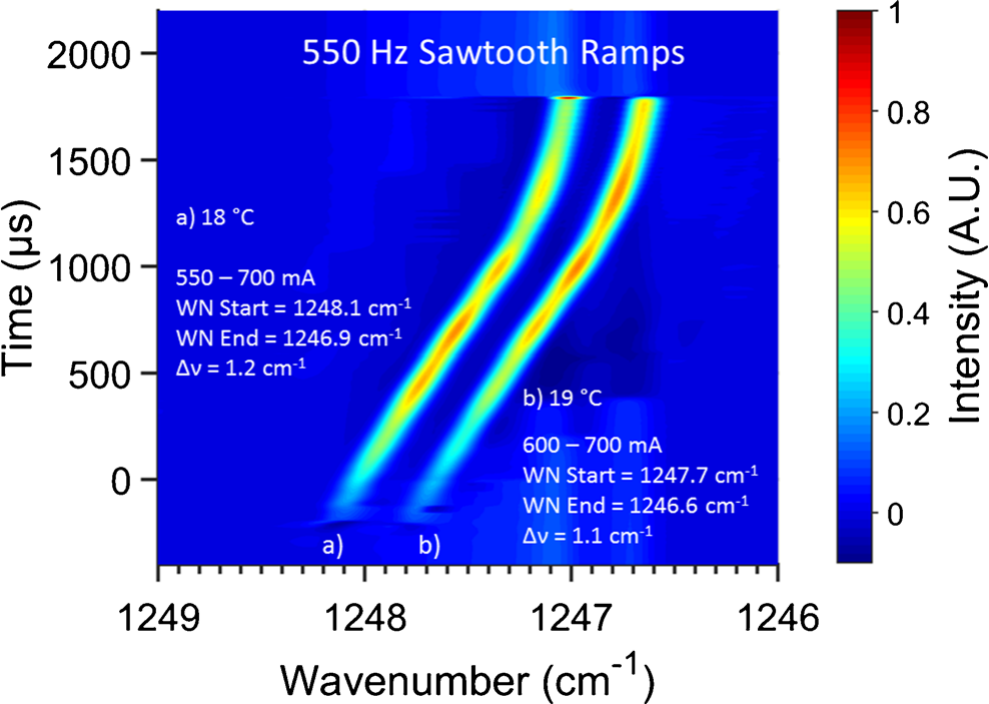
Time-resolved spectral evolution of the DFB-QCL emission during two exemplary 550-Hz sawtooth current ramps at two different laser temperatures. (**a**) For an injection current range from 550 to 700 mA and a laser temperature of 18 °C, the tuning range was 1248.1–1246.9 cm^−1^ (Δ*ν* = 1.2 cm^−1^). (**b**) For an injection current range from 600 to 700 mA and a laser temperature of 19 °C, the tuning range was 1247.7–1246.7 cm^−1^ (Δ*ν* = 1.1 cm^−1^)

The time-resolved spectra of two exemplary 550-Hz sawtooth laser current ramps at different laser temperatures ranging from 550 to 700 mA within the achievable QC laser tuning range recorded in combination with an 8-bit resolution and 500 MS/s sample rate transient recorder board (Spectrum GmbH, Germany) are shown in Fig. 1. At a laser temperature of 18 °C and injection current of 550–700 mA, the tuning range was 1248.1–1246.9 cm^−1^ (Δ*ν* = 1.2 cm^−1^), whereas at a laser temperature of 19 °C and injection current of 600–700 mA, the tuning was measured starting from 1247.7 cm^−1^ and expanding to 1246.6 cm^−1^ (Δ*ν* = 1.1 cm^−1^). Almost linear spectral evolution is observed during ∼70 % of the current ramp time scale. The non-linear spectral behavior of the last 30 % of the total ramp time scale is attributed to the onset of the safety current soft clamping (asymptotical convergence to the maximum current of 700 mA) of the laser driver.

### H_2_S sensor prototype architecture

In order to meet the on-site safety regulations, the optical platform of the H_2_S sensor was installed in an industry rack and equipped with the required safety infrastructure as suggested in the ATEX directive for protected operation in hazardous and explosive environments. The assembled H_2_S sensor prototype combines a purge and pressurization system with integrated safety electronic devices, achieving a versatile explosion prevention and malfunction protection.

**Fig. 2:**
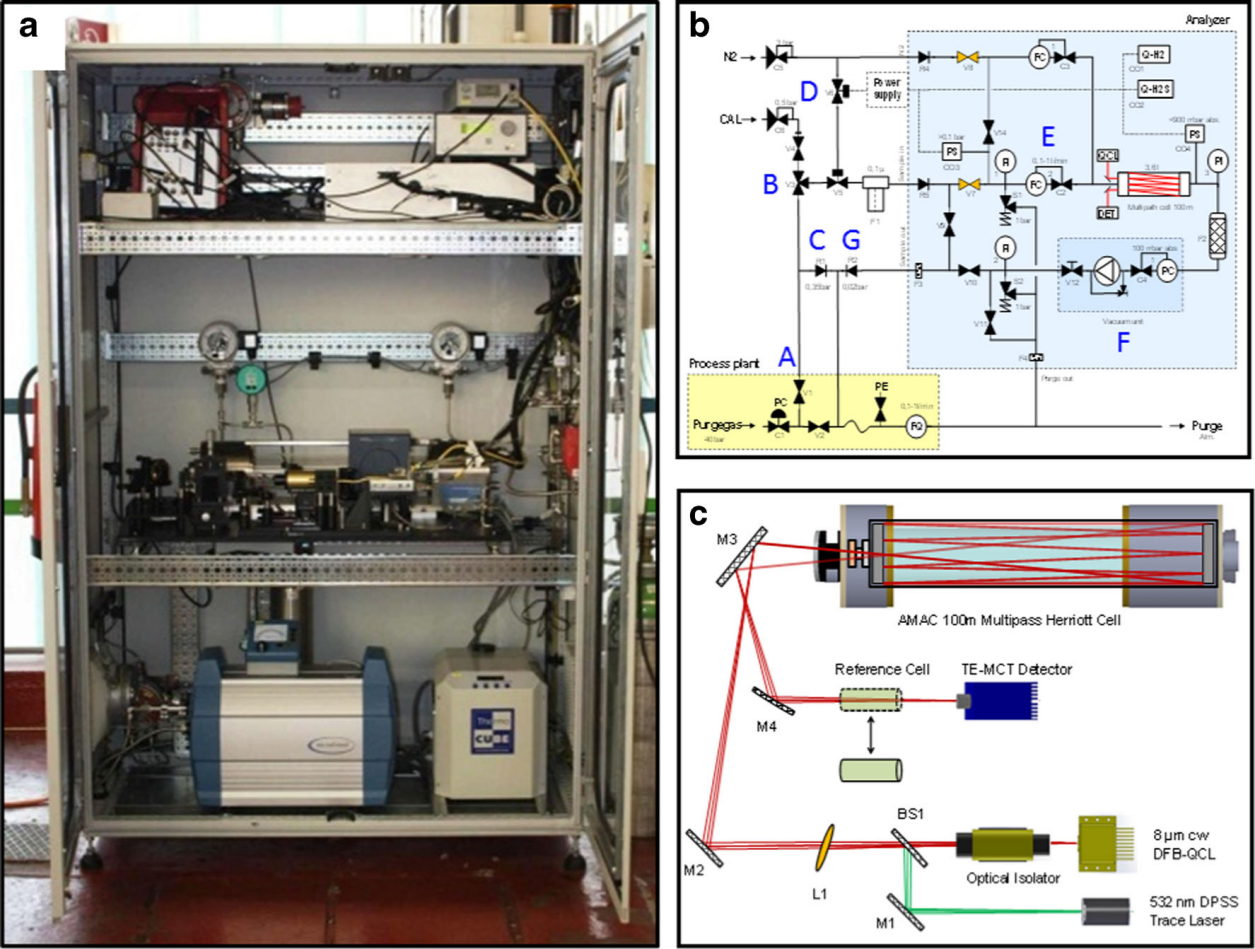
(**a**) Industry rack with the H_2_S sensor prototype and peripheral components. (**b**) Piping, instrumentation, and safety flow diagram. (**c**) Optical layout of the H_2_S sensor prototype

Figure 2a shows a photograph of the H_2_S sensor prototype fully assembled and equipped with the purge, pressurization, and integrated safety system components in the industry rack. The top floor is occupied by the driving, data acquisition (DAQ), and safety electronics. The optical platform, mass flow controllers (MFCs), pressure indicators, and valve arrays are installed in the middle floor of the industry rack. Peripheral components including the main pump, heat exchangers, and pulsation dampers are accommodated in the lower floor.

The piping, instrumentation, and safety flow diagram is depicted in Fig. 2b. The high-pressure process gas stream is sampled (A) and expanded to an intermediate pressure level (B). In case of over-pressurization, a part of the process gas stream is discharged via the back-pressure valve pathway (C). During normal operation, the safety valve (D) opens the pathway through a fine particulate filter and ensures the process gas to enter the MFC-controlled sample inlet (E) of the multipass cell. The membrane pump together with the MFCs (F) ensures constant optimal pressure levels while also maintaining a constant flow throughout the operation of the sensor prototype. Finally, the process gas stream is fed back to the purge system via a back-pressure valve (G).

The optical layout of the H_2_S sensor is outlined in Fig. 2c. The MIR laser radiation of the CW-DFB-QCL was overlaid with a visible 532-nm diode-pumped solid-state (DPSS) trace laser beam, collimated with a plano-convex lens (*f* = 500 mm) and coupled into an astigmatic Herriott multipass gas cell with a total pathlength of 100 m (AMAC100, Aerodyne Inc.). The laser wavelength was scanned at 1 Hz over the tuning range of the QCL and additionally sinusoidally modulated in the range of 1–50 kHz. This wavelength-modulated laser beam is transmitted through the absorbing, gaseous path in the multipass sample cell, giving rise to harmonic components in the optical signal. The laser radiation exiting the multipass sample cell containing the spectral information of the target analytes encoded in the modulated optical signal was focused onto an optically immersed TE cooled MCT detector (PCI-2TE-12, Vigo Systems). The recorded signals were digitized with a 16-bit 5 MS/s DAQ unit (NI USB6366, National Instruments) and further demodulated and processed using a software-implemented lock-in amplifier. Averaging of over 10 sweeps resulted in a total response time of ∼15 s.

## Results and discussion

### Laboratory-based H_2_S assessment in a CH_4_ matrix

Different H_2_S and CH_4_ concentration levels were prepared by 5.0 N_2_ (99.999 %) dilution from 2000 ppmv H_2_S- and 50,000 ppmv CH_4_-standardized gas bottles (matrix N_2_) with a mass flow and pressure-controlled in-house developed gas handling system. Pressure monitoring was performed with a digital manometer (Leo3, Keller). The multipass cell was operated at room temperature (295 ± 2.5 K) and at total pressures ranging from 50 to 100 mbar in order to spectrally resolve the ro-vibrational bands of the analyte and matrix molecules.

Driving the QCL with a current ramp ranging from 500 to 700 mA enabled a maximum spectral bandwidth of ∼1.2 cm^−1^ (Fig. 1) which allowed for the examination of multiple selected target analyte peaks in a CH_4_ matrix (compare with Table 1). The first step towards a successful multi-analyte detection was the evaluation of a selective and sensitive H_2_S assessment with the help of two reference cells. These cells, designed with an optical pathlength of 5 cm, are filled with the single-target analytes with 98 %v H_2_S and 5 %v CH_4_ backfilled with N_2_ to a total pressure of 50 mbar. They are sealed with wedged and Brewster angle-tilted CaF_2_ windows.

**Fig. 3:**
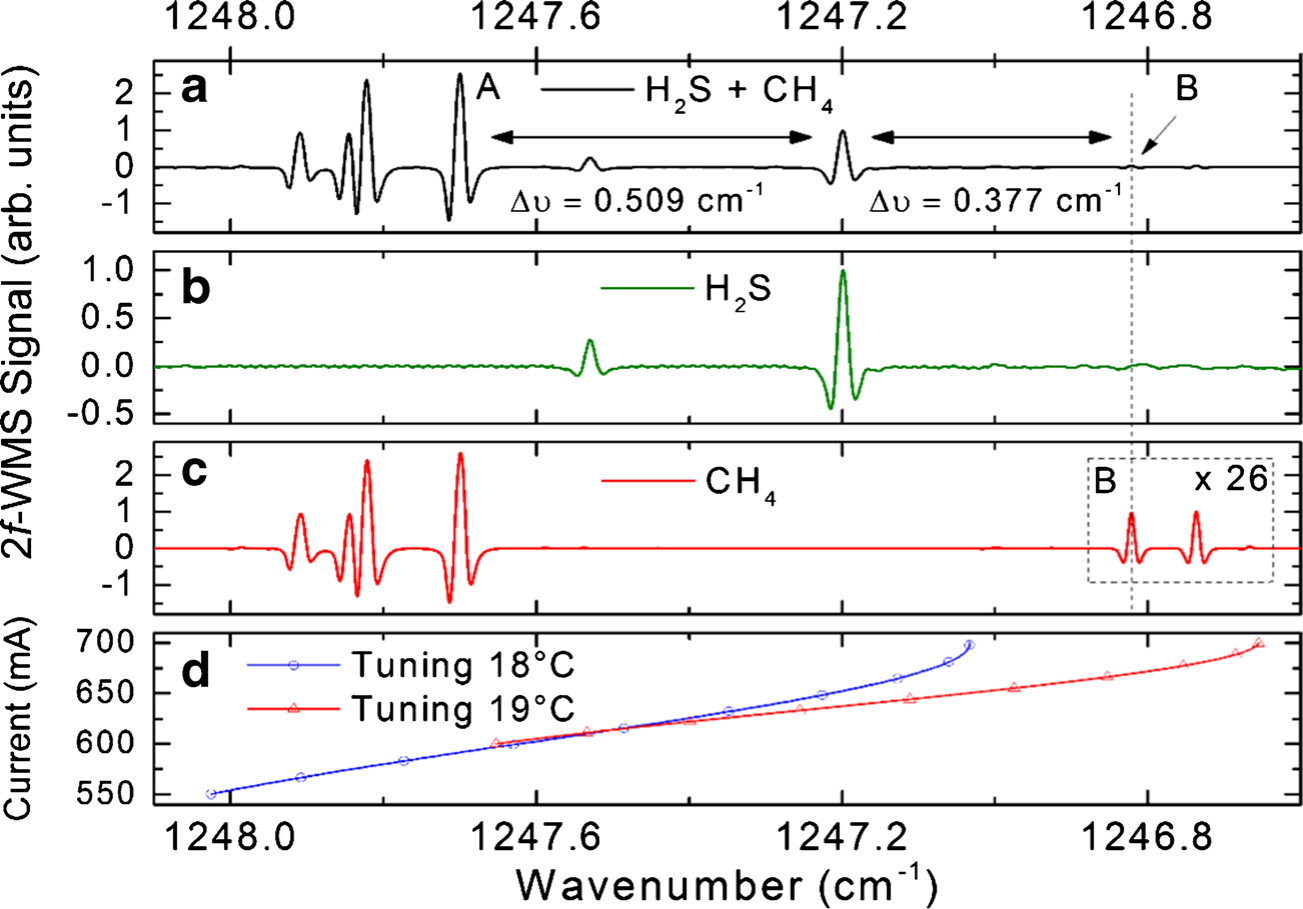
(**a**) 2*f*-WMS reference gas cell spectra with H_2_S and CH_4_ contribution. The interfering CH_4_ transitions are marked with (**A**). (**b**) 2*f*-WMS spectra of the isolated H_2_S signal contributions. Baseline ripples are due to etaloning effects of the 5-cm reference cell. (**c**) 2*f*-WMS spectra of the isolated CH_4_ signal contributions with a 26 times magnified *inset* of the interfering CH_4_ transitions (marked with (**B**)). (**d**) Tuning curves for a laser injection current ramp of 550–700 mA and a laser temperature of 18 °C (*blue*) and for an injection current ramp of 600–700 mA and a laser temperature of 19 °C (*red*)

Taking advantage of spectral line resolution at the low-pressure conditions of ∼50 mbar, a sufficient separation of Δ*ν* = 0.509 cm^−1^ from the interfering CH_4_ transitions (marked with “A”) and Δ*ν* = 0.377 cm^−1^ (marked with “B”) could be demonstrated with the two reference cells (Fig. 3). Thus, selective and interference-free H_2_S assessments in a CH_4_ matrix can be expected in an industrial matrix.

In order to assess the applicability of analytical and industrial process purposes, a synthetic CH_4_ matrix of up to 1000 ppmv together with a varying H_2_S content was prepared with the custom-built gas handling system and chosen as the model environment for subsequent measurements in the optical multipass gas cell.

**Fig. 4:**
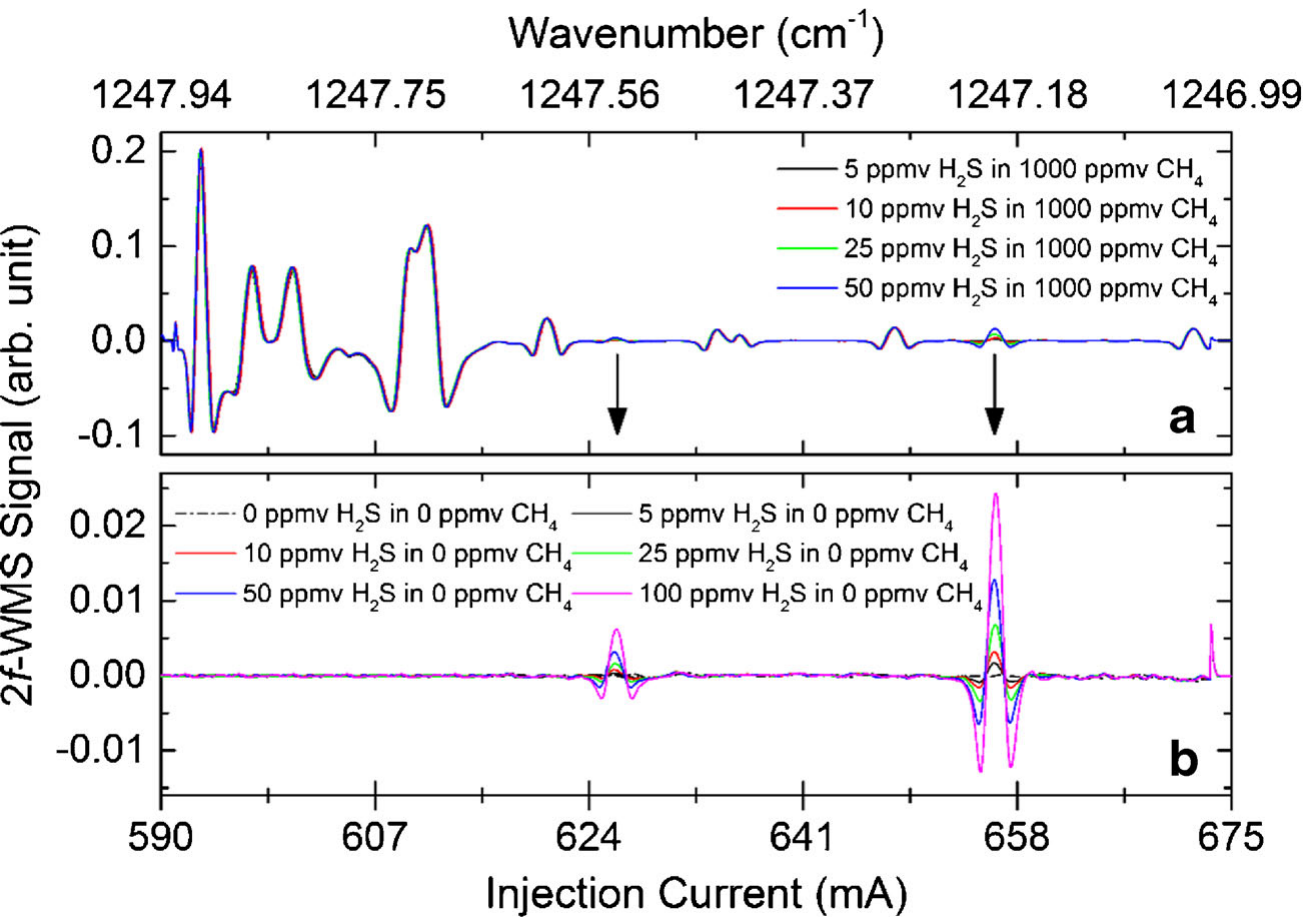
2*f*-WMS spectra of 5–50 ppmv H_2_S in a 1000 ppmv CH_4_ matrix (**a**). The isolated H_2_S contribution of 0–100 ppmv in the same tuning range is shown in (**b**)

In this context, it was possible to conduct and demonstrate selective and interference-free H_2_S assessments in a synthetic CH_4_ matrix. In Fig. 4, the according 2*f*-WMS spectra of 5–50 ppmv H_2_S in a 1000 ppmv CH_4_ matrix (Fig. 4(a)) and the isolated H_2_S contribution of 0–100 ppmv in the same tuning range (Fig. 4(b)) are shown.

### Determination of noise-equivalent absorption sensitivity and limit of detection

Quantitative measurements of H_2_S were performed using dry H_2_S gas mixtures in order to investigate the sensitivity and linear response of the 2*f*-WMS-based sensor system. A commonly accepted metric for instrument comparison is the noise-equivalent absorption sensitivity (NEAS), which can be described as the minimum detectable absorption scaled to pathlength and noise-equivalent detection bandwidth [11, 39]

2 $$ \mathrm{NEAS}=\left(\frac{\varDelta I}{I}\right)\left(\frac{1}{L\sqrt{\mathrm{BW}/N}}\right) $$

where Δ*I*/*I* is the 1*σ* value of the limiting noise level in the spectrum, normalized by the total intensity (*I*); *L* is the optical pathlength; BW is the detection bandwidth; and *N* is the number of averages.

**Fig. 5:**
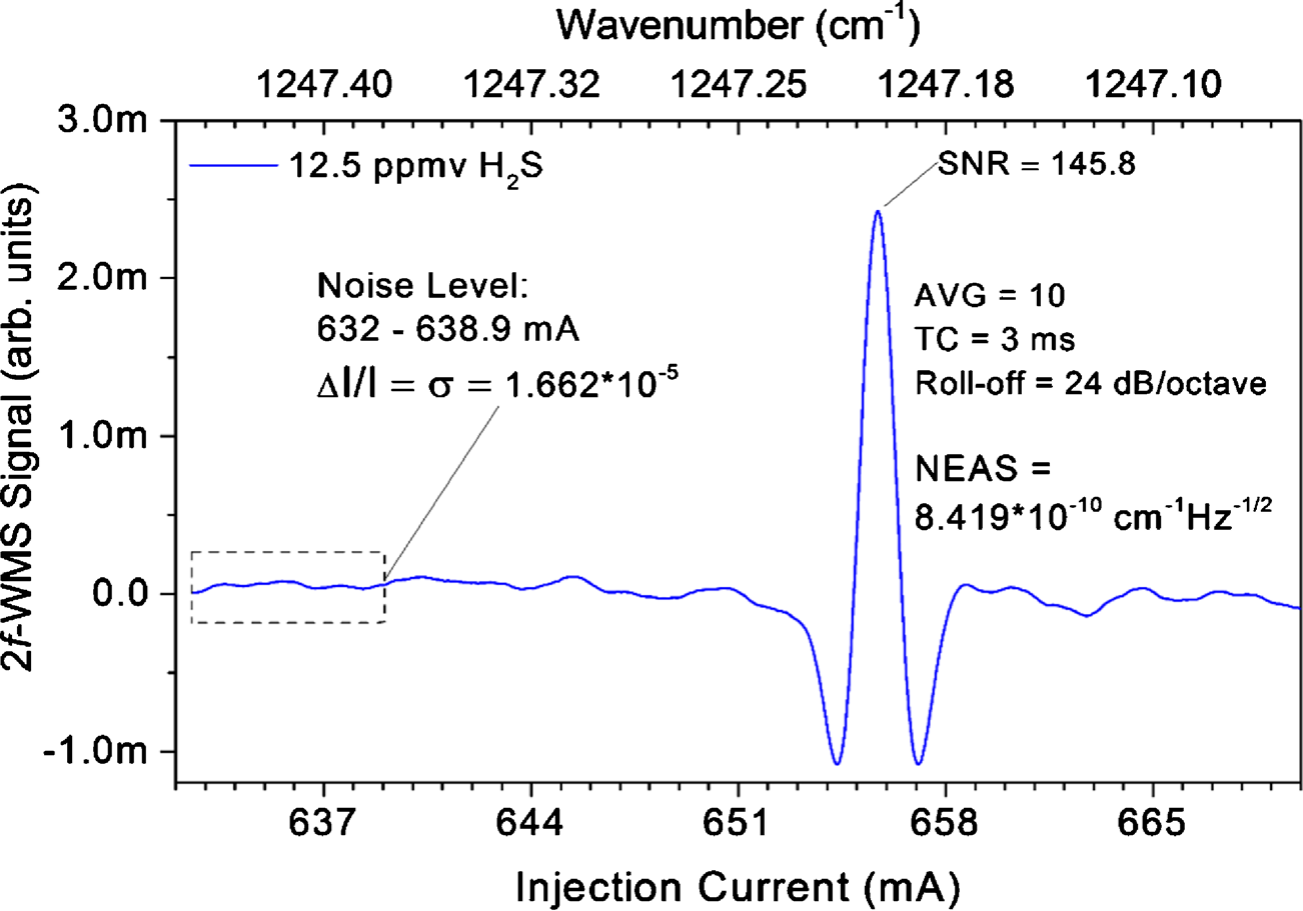
Determination of the noise-equivalent absorption sensitivity (*NEAS*) of H_2_S

For the NEAS determination, 2*f*-WMS spectra of 12.5 ppmv H_2_S at 65 mbar were acquired with a lock-in time constant of 2 ms, a filter slope (roll-off) of 24 dB/octave, and averaged 10 times which resulted in a noise-equivalent bandwidth of the low-pass filter of 39 Hz (refer to Fig. 5). The estimated value of the NEAS for H_2_S at ∼1247.2 cm^−1^ was found to be 8.419 × 10^−10^ cm^−1^ Hz^−1/2^.

The 2*f*-WMS technique measures a signal roughly proportional to the second derivative of the scanned absorption line shape, hence decreasing the vulnerability to slow baseline drifts and effectively rejecting 1/*f* noise. In comparison to direct absorption spectroscopy approaches, instrument sensitivity is typically improved by one order of magnitude. Due to the more complex nature of the measured signals, the implementation of 2*f*-WMS reference spectra for derived concentration measurements requires elaborate analytical or numerical simulation of taking crucial parameters of laser wavelength, optical power, modulation depth, absorption line shape, residual amplitude modulation (RAM) of the laser power, and detector response into account. In practice, the majority of 2*f*-WMS-based trace gas sensors are preferred to be calibrated using reference calibration gas mixtures [40–42], despite the availability of precise modeling of 2*f*-WMS spectra [22, 43, 44].

**Fig. 6:**
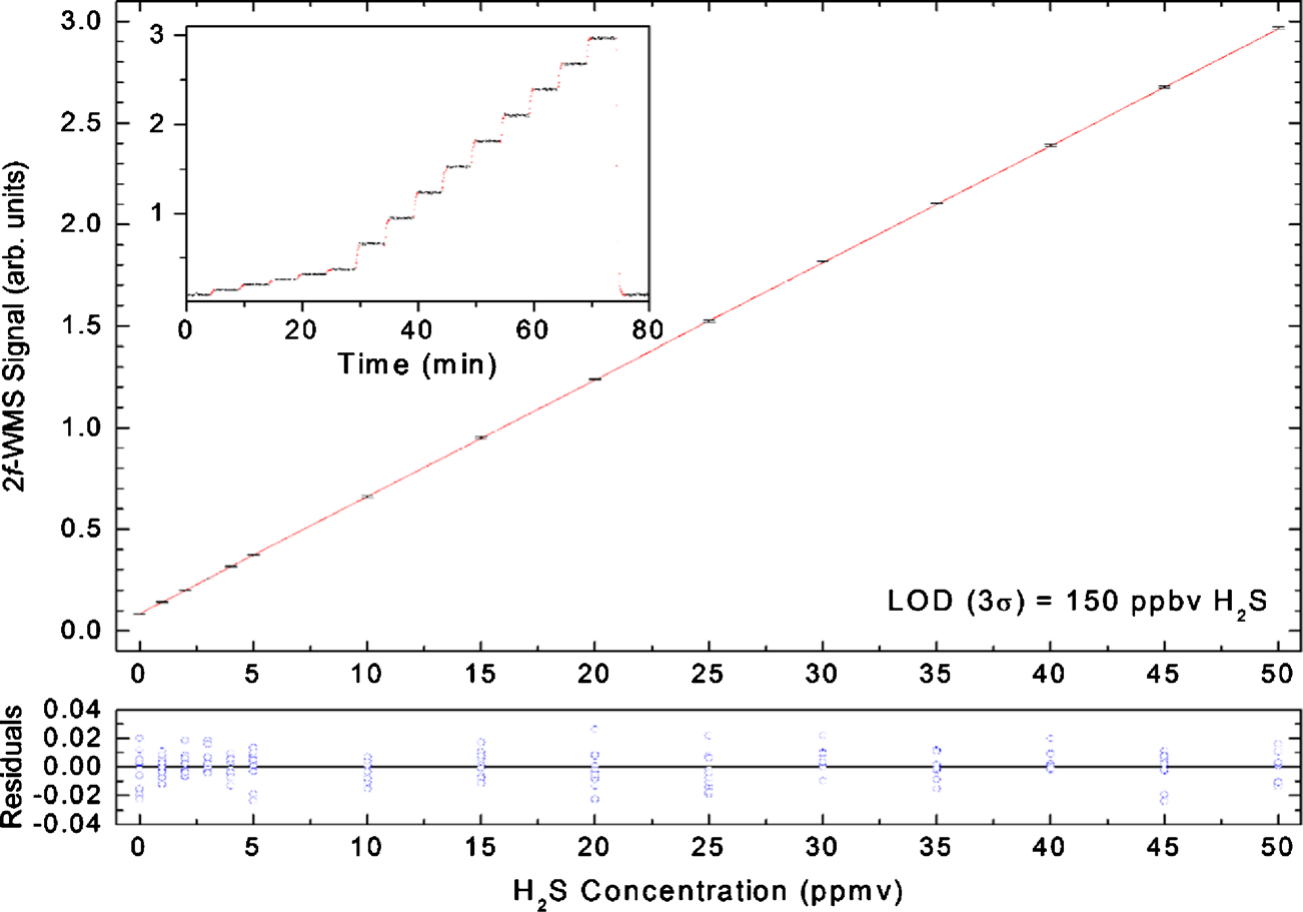
Calibration curve of 0–50 ppmv H_2_S. The calculated LOD (3*σ*) is 150 ppbv H_2_S

For the evaluation of the limit of detection (LOD) of the 2*f*-WMS sensor prototype, different H_2_S concentration levels within a range from 0 to 50 ppmv were prepared by diluting a certified 100 ppmv H_2_S:N_2_ calibration mixture with 5.0 N_2_ (99.999 %). Each concentration step was measured 10 times, and the resulting data were averaged and plotted as a function of concentration (refer to Fig. 6). The good linearity between signal amplitudes and H_2_S concentrations was observed for the 2*f*-WMS-based sensor. The corresponding LOD was ascertained with the Validata software package [45] at three times the standard deviation (3*σ*) of the intercept divided by the slope of the calibration curve, which resulted in 150 ppbv.

### On-site results

Results from online measurements at the project partner OMV AG are depicted in the following figures. The exemplary data is typically plotted over several hours to several days. Absolute values of concentration scales and additional plant parameters (catalyst compositions, temperature, and pressure levels) are omitted due to company regulations. However, a general description of the fundamental process is given.

The H_2_S concentration is derived from the correlation of the measured 2*f*-WMS spectra with the reference spectra of a precisely validated gas standard, acquired during the calibration procedure. With this calibration technique, high-precision gas concentration measurements can be performed, as the absolute accuracy of the instrument calibration is predominantly related to the precision of the applied certified gas standard. Moreover, this approach does not rely on the determination of any of the previously stated parameters, which are required for 2*f*-WMS spectral simulation [22, 46–48].

Although no explicit calibration curve was recorded for CH_4_, the concentration was calculated by applying the deduced H_2_S sensitivity to the measured 2*f*-WMS signal of the CH_4_ transitions centered around 1246.8 cm^−1^, as these CH_4_ transitions exhibit similar linestrengths compared to the H_2_S feature at 1247.2 cm^−1^.

**Fig. 7:**
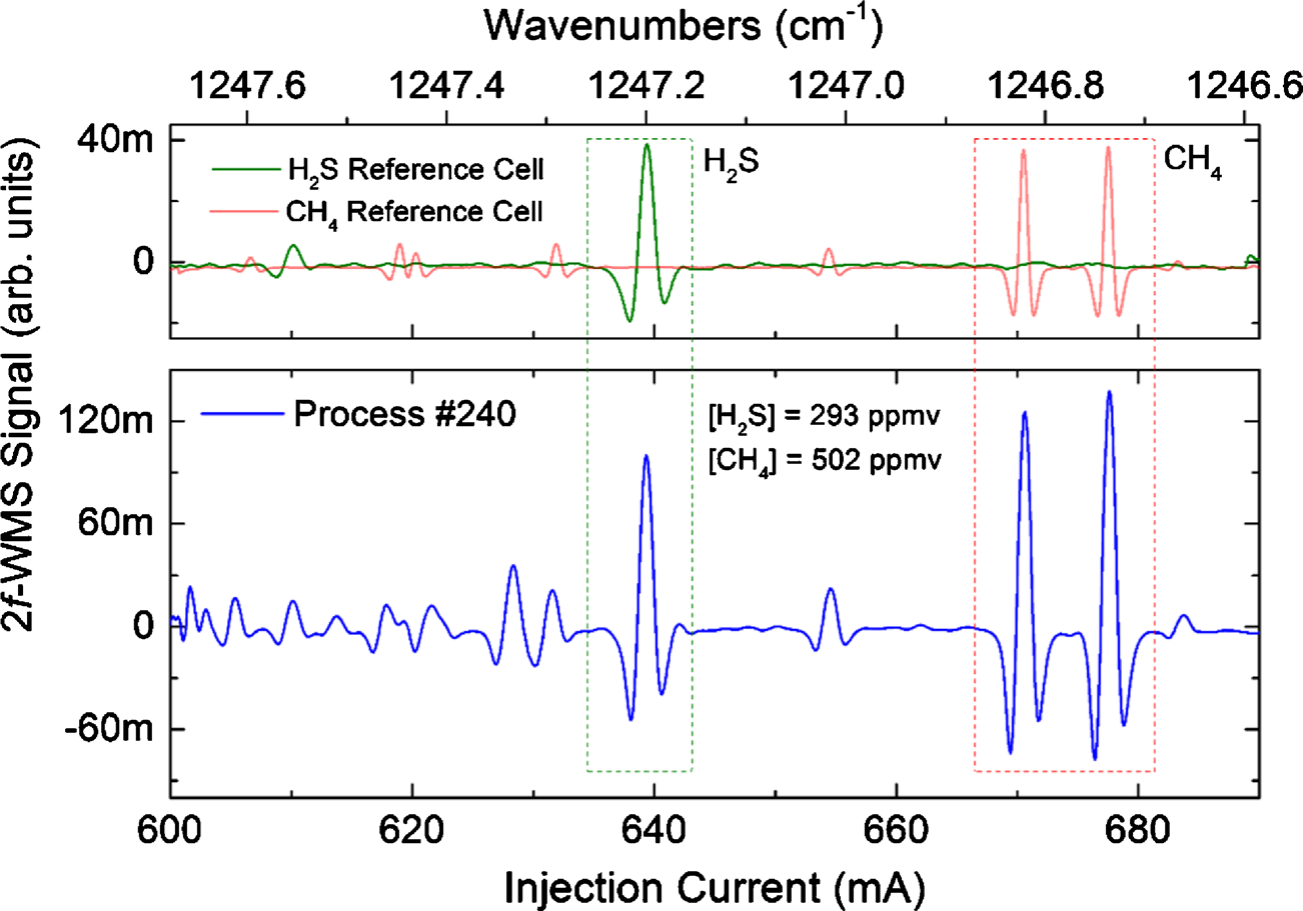
Exemplary process spectrum of a HDS run. The positions of the H_2_S and CH_4_ features are marked

An exemplary online purge gas process spectrum of the hydration reaction plant containing ∼300 ppmv H_2_S and ∼500 ppmv CH_4_ together with the referenced analyte positions is shown in Fig. 7.

**Fig. 8:**
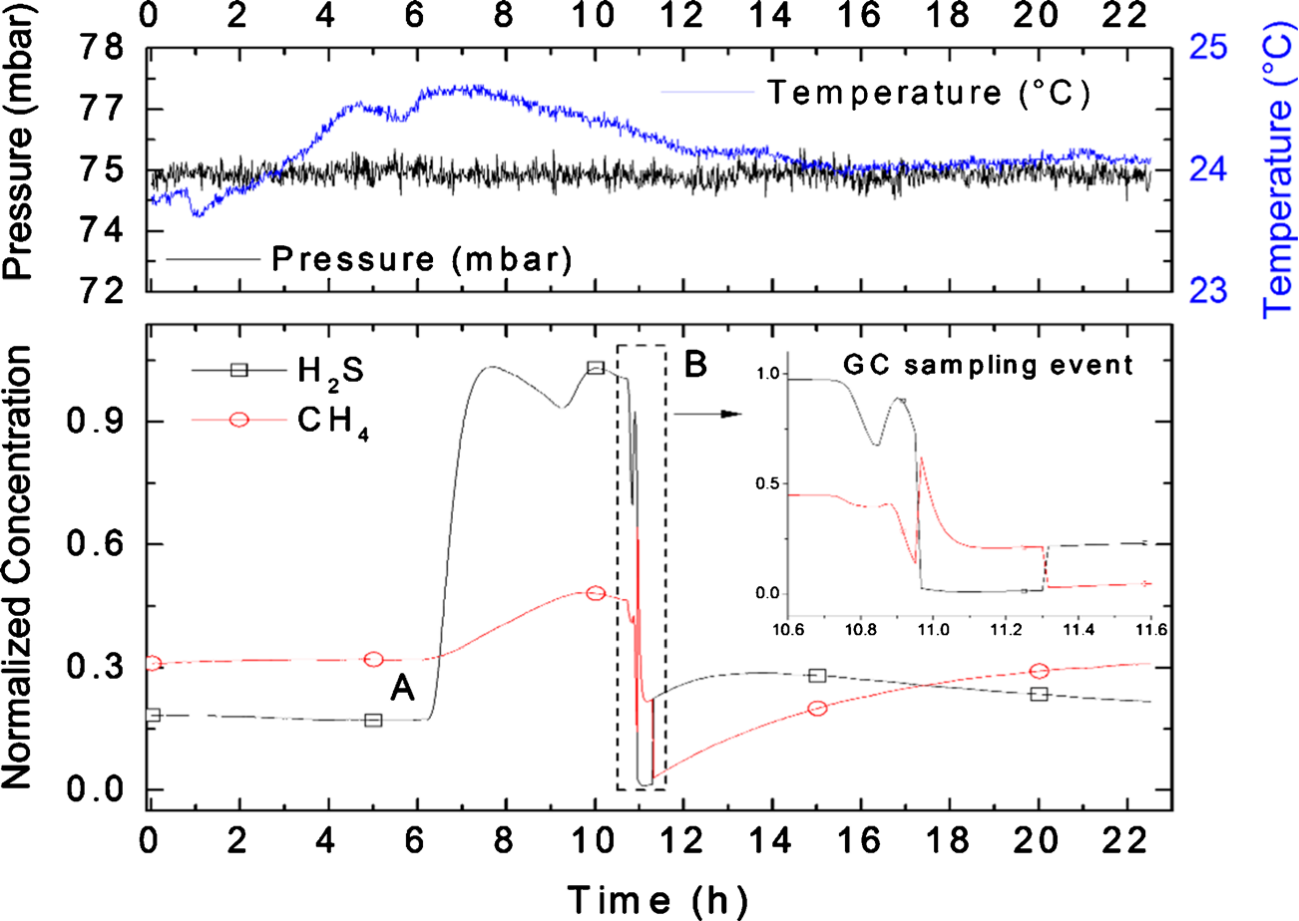
Continuous monitoring of the H_2_S and CH_4_ content during a 22-h HDS run. The transient reactor response due to a GC sampling event could be revealed by the fast sensor response

The continuous monitoring of the H_2_S and CH_4_ content during a 65-h-lasting HDS run of straight-run oil is visualized in Fig. 8. The defined feed change event at *t* = 6 h (marked with A) as well as the transient HDS reactor response due to a GC sampling event at *t* = 10.7 h (marked with B) could be revealed by the fast sensor response.

**Fig. 9:**
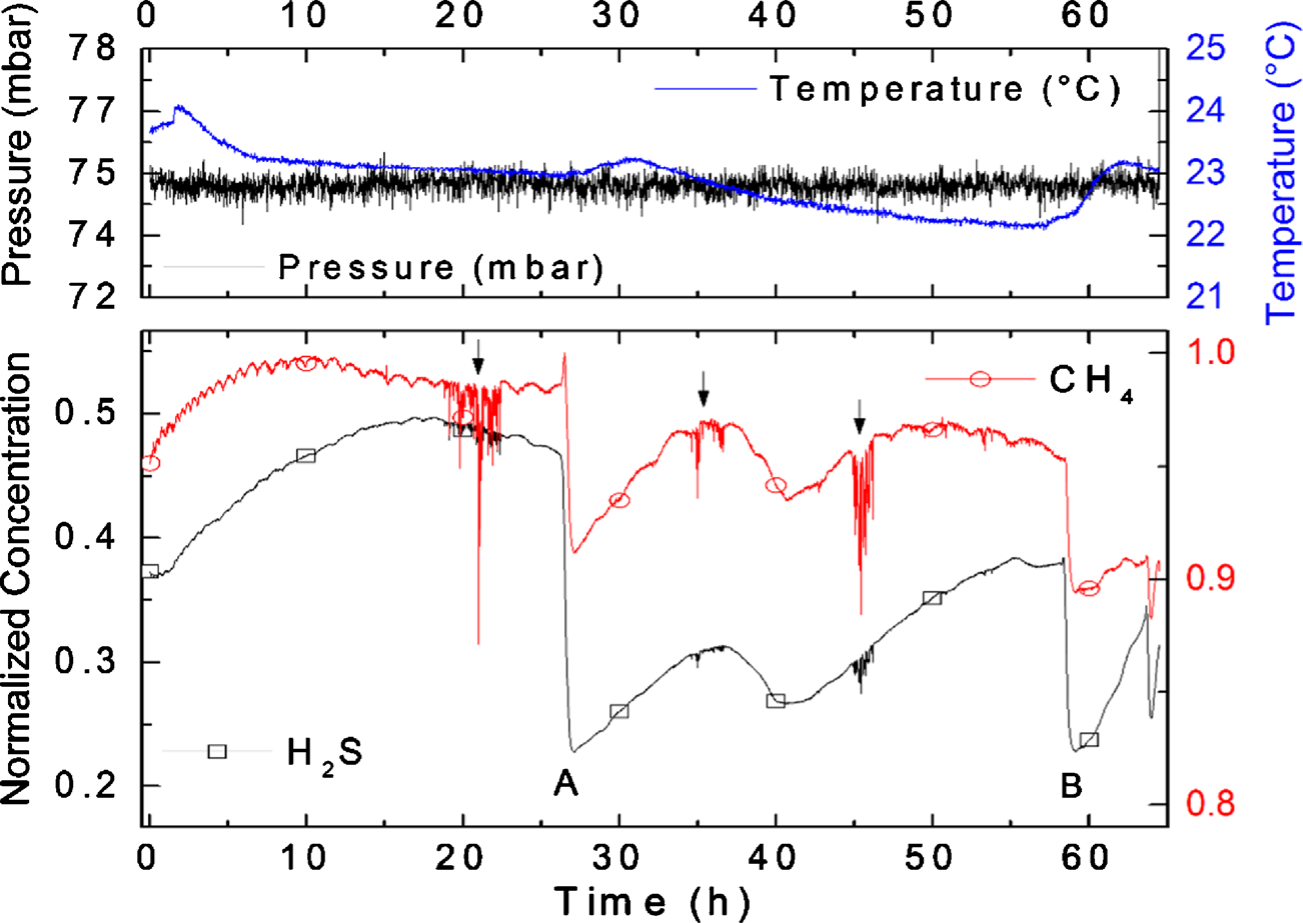
Continuous monitoring of the H_2_S and CH_4_ content during a 65-h HDS run. At points (**A**) and (**B**), the feedstock was changed. Interesting to note is the periodic fluctuation of the CH_4_ content during the first 15 h of operation. Transient reactor dynamics resulting in concentration dips due to likely upsets and instabilities in the flow rates at *t* = 20 h, *t* = 30 h, and *t* = 45 h are marked by *arrows*

The continuous monitoring of the H_2_S and CH_4_ content during a 65-h-lasting HDS run of low-sulfur feedstocks is visualized in Fig. 9. The defined feed change events at *t* = 26 h (marked with A) and *t* = 58 h (marked with B) are indicated. During the first 15 h of operation, an interesting and still unexplained effect of the periodic fluctuation of the CH_4_ content was observed. Moreover, transient reactor dynamics resulting in concentration dips due to likely upsets and instabilities in the flow rates at *t* = 20 h, *t* = 30 h, and *t* = 45 h could be discovered during operation.

## Summary and conclusions

A MIR optical gas sensor prototype based on wavelength modulation spectroscopy with second harmonic detection (2*f*-WMS), employing a continuous-wave distributed feedback quantum cascade laser (CW-DFB-QCL) emitting at 8 μm, for fast, sensitive, and selective sub-ppmv H_2_S detection was developed.

In order to assess the applicability for analytical and industrial process purposes aimed at petrochemical environments, a synthetic methane (CH_4_) matrix of up to 1000 ppmv together with a varying H_2_S content in an optical 100-m multipass gas cell was chosen as the model environment. A noise-equivalent absorption sensitivity (NEAS) for H_2_S at ∼1247.2 cm^−1^ was found to be 8.419 × 10^−10^ cm^−1^ Hz^−1/2^. In the same spectral region, a limit of detection (LOD) of 150 ppbv H_2_S could be achieved.

Subsequently, a sensor prototype was developed, installed in an industry rack, and equipped with the required safety infrastructure for protected operation in hazardous and explosive environments, in order to meet the company on-site safety regulations. The sensor prototype was deployed and successfully tested for on-site measurements under the imperative on-site safety regulations for hazardous and explosive environments at the petrochemical research hydrogenation platform of the industrial partner OMV AG.

In comparison with the industrially established reference GC method, the sensor prototype clearly acted as a new tool for monitoring the H_2_S content with faster response times and allowed to access additional CH_4_ concentration information.

Important analytical process advantages of the developed H_2_S sensor prototype are identified in its high selectivity paired with sub-ppmv sensitivity and fast response time, which allow a continuous, direct pre-treatment-free measurement of the process gas streams.

Clearly, a major challenge for the industrial implementation of QCL technology is finding a precisely tailored application with recognizable advantages while exhibiting a pronounced cost benefit for using QCLs. For spectroscopic applications, the MIR spectral region is favored over the near-infrared region due to the considerably stronger cross sections of the chemicals under investigation. But, it is much less desirable when considering the availability and system costs of the expensive optical components and materials. As a consequence, it is believed that the QCL technology will start to penetrate industrial markets, where process gas detection is deemed necessary and no other solutions with current techniques are available.

Planned research will benefit from the generic nature and flexibility of the QCL-based sensor classes. Implementation of new QCL designs, such as multiple DFB chips [49–51], RCSE arrays [52], or Vernier-effect QCLs [53, 54], will allow for a further extended spectral coverage. Thus, other ro-vibrationally accessible analytes of particular interest can be measured as well. In addition, integration of laser source and detectors in one device is possibly opening the path for highly miniaturized, sensitive gas sensors for detecting multiple gas species [55, 56].

Further development and extension of the integrated safety and malfunction protection infrastructure in order to achieve a certified ATEX status will assure proper operation under the mandatory petrochemical safety regulations and prepare the ground for true on-site applicability of the QCL-based sensor prototype.
